# Supplementary material for: Mapping cell-to-tissue graphs across human placenta histology whole slide images using deep learning with HAPPY
Source: Nat Commun. 2024 Mar 28;15:2710. doi: 10.1038/s41467-024-46986-2 (PMC10978962; doi:10.1038/s41467-024-46986-2)
Supplement: Supplementary file 1 — Supplementary Information [file 41467_2024_46986_MOESM1_ESM.pdf]

Supplementary Information

Supplementary Figures 1-8

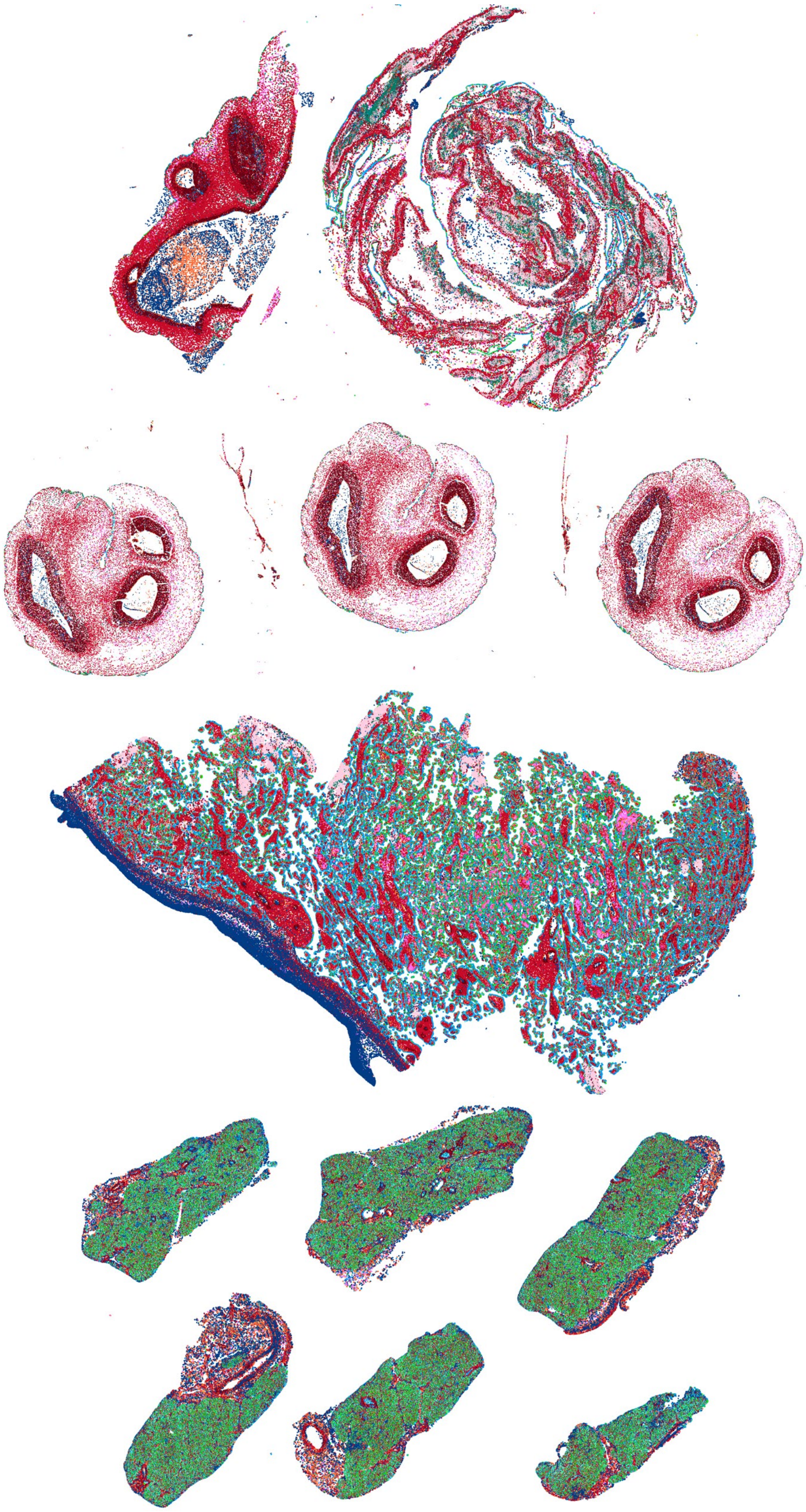

Supplementary Fig. 1: Visualisation of a preliminary exploration of applying nuclei localisation and cell classification models across other placenta cuts and other organs. The predictions of nuclei localisation and cell classification models trained for placenta parenchyma histology applied to healthy term placenta membrane rolls (a), healthy term placenta umbilical cords (b), second trimester placental parenchyma with chorioamnionitis (c) from our in-house data and (d) pancreas samples from the GTEx dataset.

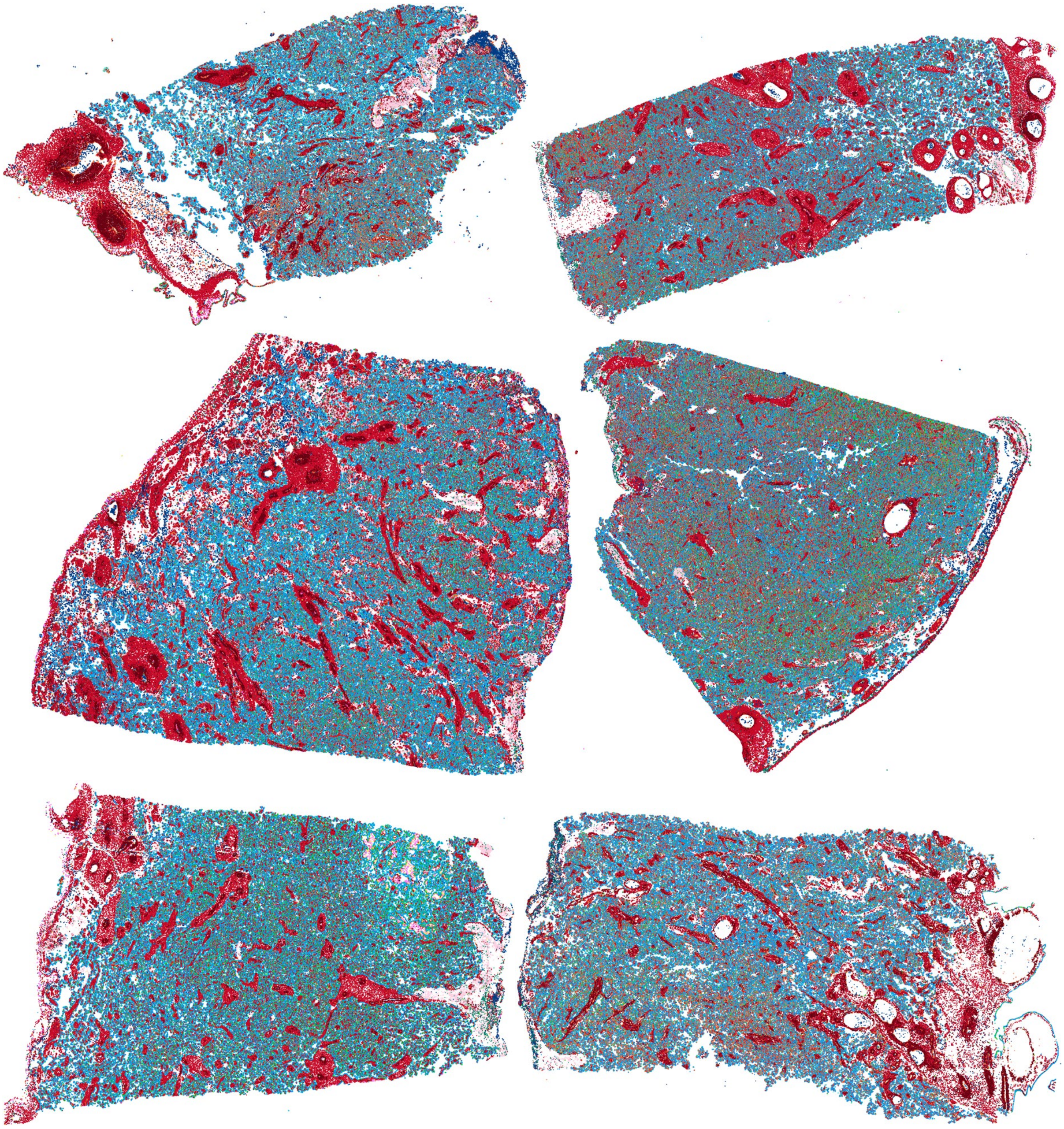

Supplementary Fig. 2: Visualisation of nuclei and cell predictions across six WSIs of healthy term placenta parenchyma from three institutes.

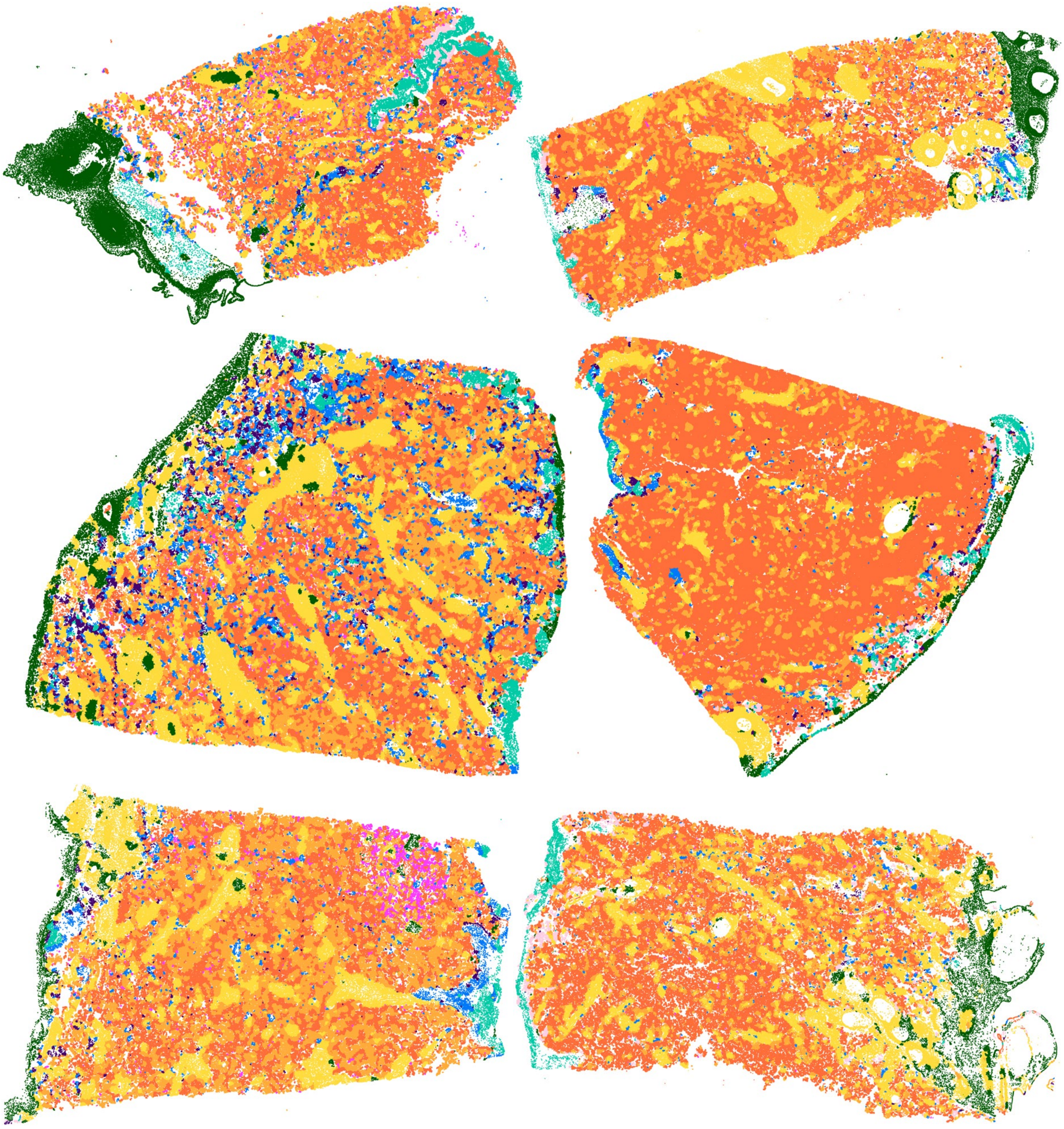

Supplementary Fig. 3: Visualisation of tissue node predictions across six WSIs of healthy term placenta parenchyma from three institutes.

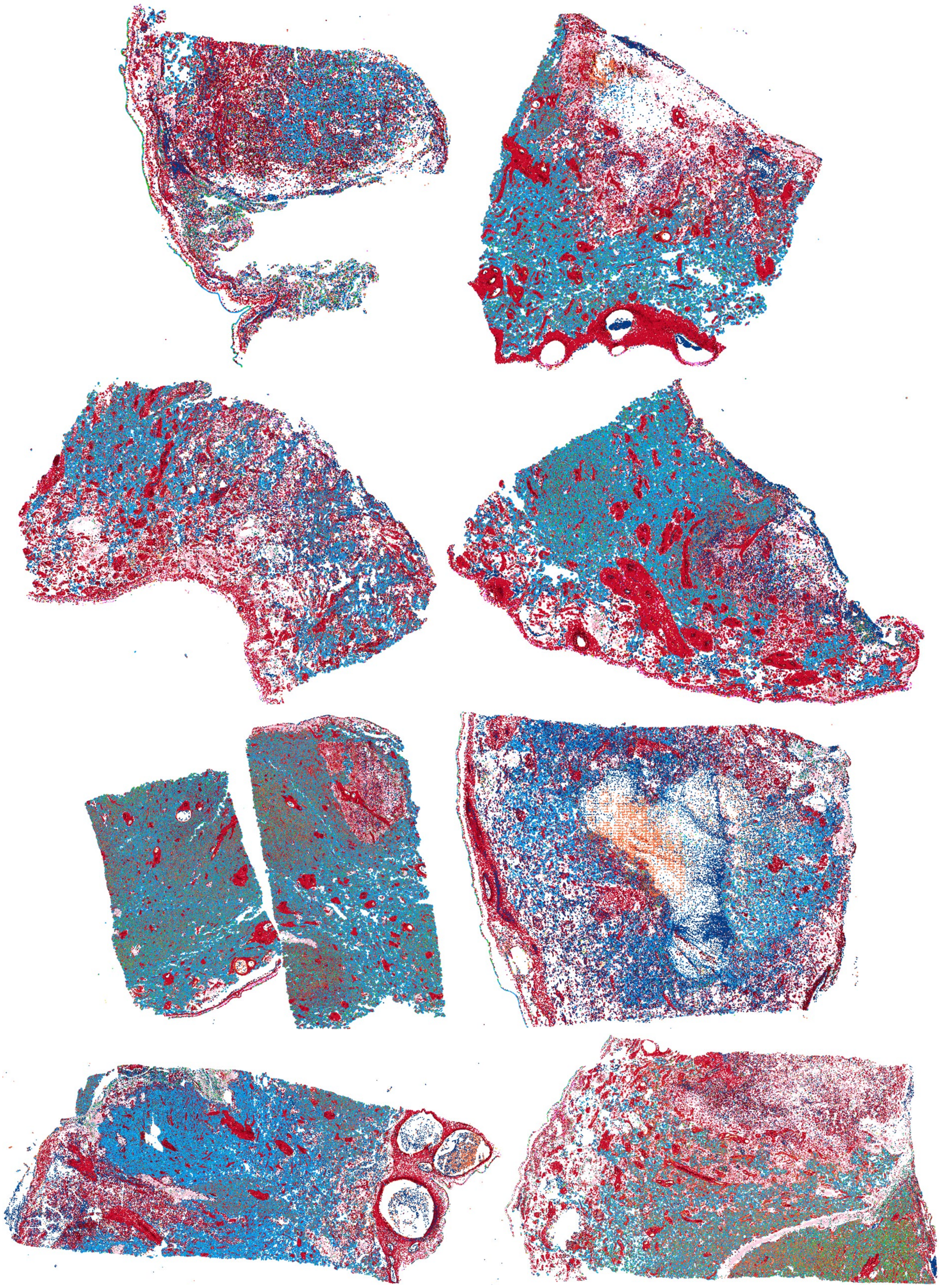

Supplementary Fig. 4: Visualisation of nuclei and cell predictions across eight WSIs of term placenta parenchyma with clinically significant placental infarction.

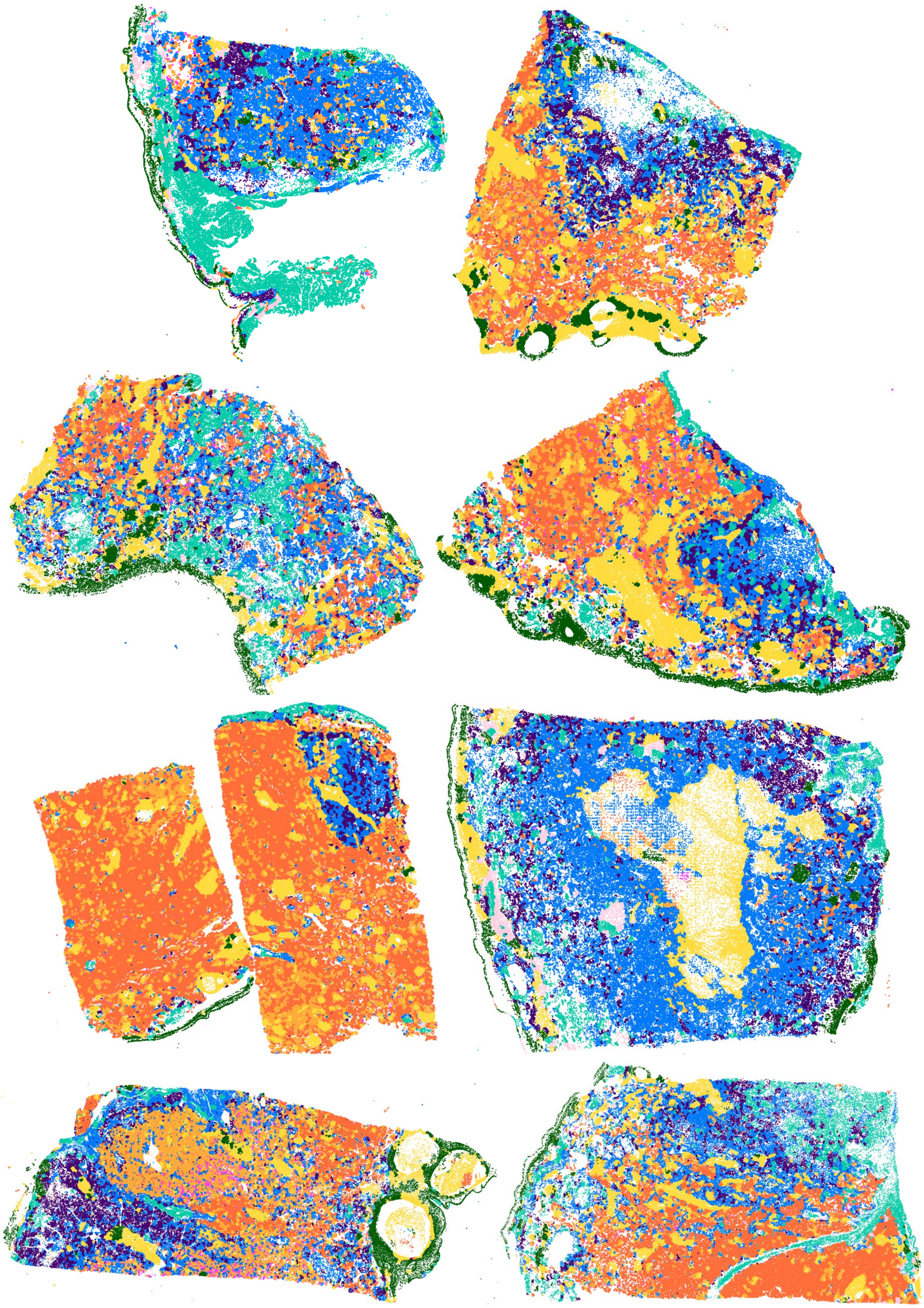

Supplementary Fig. 5: Visualisation of tissue node predictions across eight WSIs of term placenta parenchyma with clinically significant placental infarction.

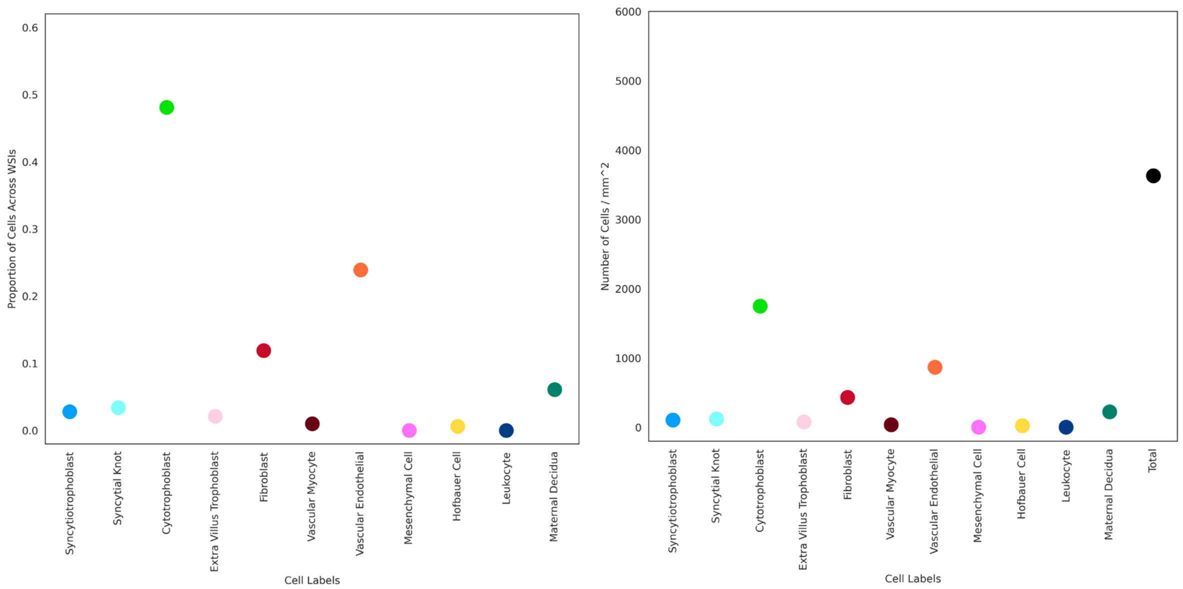

Supplementary Fig. 6: Predicted cell proportions and densities across a whole slide image (WSI) with substantially different hematoxylin and eosin staining than the WSIs the model was trained with. The staining difference causes a domain shift which results in biologically implausible cell predictions across a slide. In this case, this is apparent by the large proportion of cytotrophoblast cells. Source data are provided as a Source Data file.

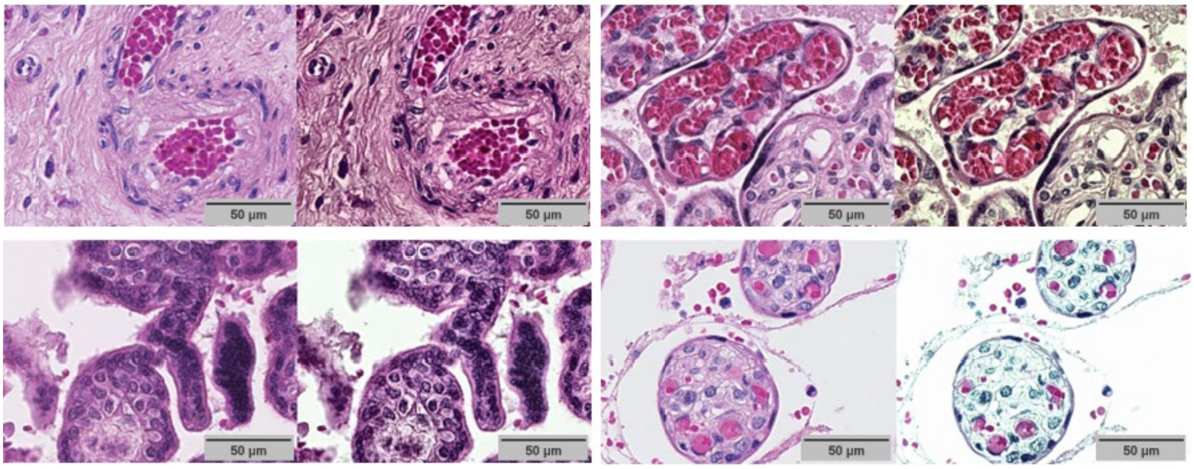

Supplementary Fig. 7: Examples of hematoxylin and eosin stain augmentation applied to nuclei localisation training data. Each image pair contains the original histology image (left) and the stain augmented histology image (right). Images are 1600x1200 (177.44x133.08 *μm*) pixel dimensions.

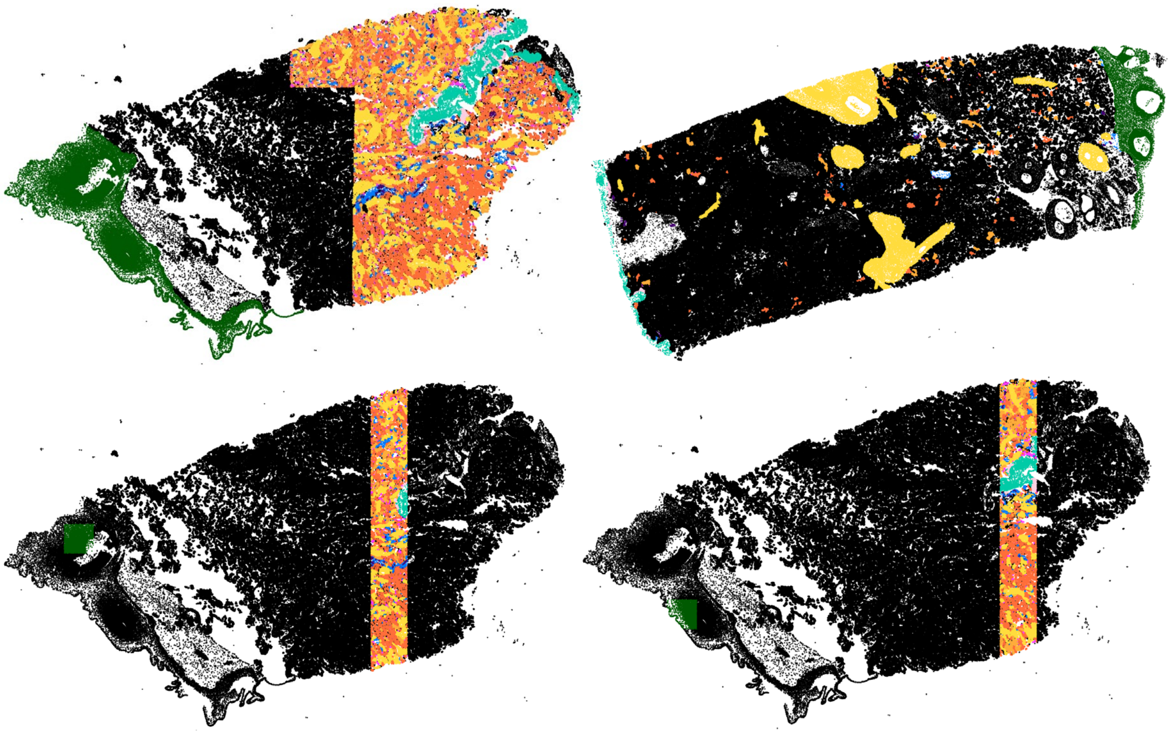

Supplementary Fig. 8: Tissue node classification labelled data and validation and test split regions of the whole slide images (WSIs). All labelled ground truth tissue node classification data across two WSIs are shown in colour with unlabelled nodes in black (top). Validation (bottom left) and test (bottom right) split regions with the remaining labelled data used for training. Regions were chosen to be larger than the 16-hop aggregation distance and to contain a similar tissue type distribution to the training set and rest of the slide.

Supplementary Tables 1-7

Table 1: Term chorionic villus characteristics.

**Villus Type**	**Diameter (*µm*)**	**Trophoblast**	**Stroma**	**Vessels/capillary density**
**Villus Sprout**	60–100	Syncytiotrophoblasts with a core of cytotrophoblasts	None or some reticular stroma	Unvascularised
**Terminal villi**	60	Vasculosyncytial membranes and syncitial knots	Scant fibrous stroma	Capillaries at more than 50% of the stromal area
**Mature Intermediate Villi**	80–150	Fully surrounded by syncytiotrophoblasts, half surrounded by cytotrophoblasts	Fibrous stroma	Capillaries at less than 50% of the stromal area
**Stem Villi**	80–3000	Thick layer, frequently replaced by fibrinoid	Fibrous stroma	Large muscularized arteries and veins, few capillaries
**Anchoring Villi**	80-3000	None or the same as stem villi	The same as stem villi	Unvascularised or the same as stem villi

Characteristic and defining cellular and morphological traits of the term chorionic villus structures. Adapted from [1,2].

Table 2: The effect of stain augmentation on nuclei detection model generalisability.

Model Type	Seen Institute	Unseen Institute	Unseen Institute
**UoT with augs**	**0.895 UoT**	0.868 HMC	**0.883 NUH**
**UoT without augs**	0.891 UoT	**0.883 HMC**	0.823 NUH
**HMC with augs**	**0.872 HMC**	0.851 UoT	**0.873 NUH**
**HMC without augs**	0.856 HMC	**0.86 UoT**	0.836 NUH
**NUH with augs**	**0.857 NUH**	**0.811 UoT**	**0.824 HMC**
**NUH without augs**	0.831 NUH	0.748 UoT	0.795 HMC

Nuclei detection model F1 score when trained with and without augmentations on training data from one institute (seen institute) and evaluated on test data from the two other institutes (unseen institute). All F1 scores are recorded over test data. Pairs of rows show corresponding results with and without augmentations with the best result in each category and pair are highlighted in bold. Source data are provided as a Source Data file.

Table 3: The effect of stain augmentation on cell classification model generalisability.

Model Type	Seen Institute	Unseen Institute	Unseen Institute
**UoT with augs**	0.978 UoT	**0.871 HMC**	**0.844 NUH**
**UoT without augs**	**0.981 UoT**	0.791 HMC	0.720 NUH
**HMC with augs**	0.971 HMC	**0.857 UoT**	**0.875 NUH**
**HMC without augs**	**0.972 HMC**	0.794 UoT	0.697 NUH
**NUH with augs**	0.959 NUH	**0.791 UoT**	**0.808 HMC**
**NUH without augs**	**0.964 NUH**	0.544 UoT	0.596 HMC

Cell classification ROC AUC when trained with and without augmentations on training data from one institute (seen institute) and evaluated on test data from the two other institutes (unseen institute). All ROC AUC results are recorded over test data. Pairs of rows show corresponding results with and without augmentations with the best result in each category and pair are highlighted in bold. Source data are provided as a Source Data file.

Table 4: Patient characteristics for each placenta used for training.

Institute	Models	Gestational Age	Relevant Clinical Data
UoT	Nuclei and Cell	30+5	Acute chorioamnionitis, infarction
UoT	Nuclei and Cell	30+5	Acute chorioamnionitis, infarction
UoT	Nuclei and Cell	40+2	Umbilical cord knot, focal acute subchorionitis, intervillous thrombosis
UoT	Nuclei and Cell	20+4	Acute chorioamnionitis, inflammation of the umbilical cord
UoT	Nuclei and Cell	42+0	Placenta vallate, hypocoiled cord
HMC	Nuclei and Cell	36+5	Suspected placenta accreta
HMC	Nuclei and Cell	41+5	Suspected placenta accreta
NUH x 2	Nuclei and Cell	37 – 42 (term)*	Histologically normal
UoT	Tissue	42+0	Placenta vallate, hypocoiled cord
HMC	Tissue	40+0	Suspected placenta accreta

Patient characteristics and relevant clinical data for each placenta histology slide from which images were used for training. Slides used to train the nuclei and cell models have a range of pathologies and gestational ages. Slides used to train the tissue model are from healthy term placentas. * Clinical data and exact gestational age for slides from NUH are undisclosed as per data transfer agreement.

Table 5: Patient characteristics for each placenta used for inference.

Institute	Gestational Age	Relevant Clinical Data
UoT	42+0	Placenta vallate, hypocoiled cord
HMC	40+0	Suspected placenta accreta
HMC	36+5	Suspected placenta accreta
HMC	40+1	Suspected placenta accreta
HMC	41+5	Suspected placenta accreta
NUH x 25	37 – 42 (term)*	Histologically normal
UoT	40+5	Infarction, intervillous thrombosis
HMC	40+4	Infarction, umbilical vessel inflammation
HMC	40+0	Infarction, intervillous thrombosis
HMC	40+1	Infarction, perivillous fibrin
HMC	41+1	Infarction, perivillous fibrin, accreta
HMC	40+4	Infarction, chorioamnionitis
HMC	40+6	Infarction, perivillous fibrin, avascular villi, chronic villitis, accreta
HMC	41+1	Infarction, perivillous fibrin, intervillous thrombosis, avascular villi, chronic villitis, accreta

Patient characteristics and relevant clinical data for each placenta used for inference. Slides from the first five placentas and 25 placentas from NUH correspond to the healthy group presented in the results. Slides from the last eight placentas correspond to the group with clinically significant placental infarction. * Clinical data and exact gestational age for slides from NUH are undisclosed as per data transfer agreement.

Table 6: Training augmentations for nuclei localisation and cell classification.

Augmentation	Probability	Additional Parameters
Flip	0.5	-
RandomRotate90	0.5	-
StainAugment	0.9	variance=0.4
CLAHE	0.7	clip limit=3.0, tile_grid_size=(8, 8)
RandomToneCurve	0.8	-
RandomBrightnessContrast	0.8	brightness_limit=(-0.1, 0.2), contrast_limit=(0.0, 0.0), brightness_by_max=False
GaussNoise	0.8	var_limit=(10.0, 200.0)
Blur	0.8	blur_limit=5

Augmentations are applied sequentially using the Albumentations library[3]. StainAugment is a custom stain deconvolution method which varies hematoxylin and eosin (H&E) stain intensities using one of 8 randomly chosen RGB to H&E colour matrices[4]. Colour matrices are drawn from the original literature and derived from our own slides.

Table 7: Datasets and Dataset Splits.

Nuclei Localisation			
	Train	Validation	Test
Nuclei	11755 (70%)	2374 (14%)	2754 (16%)
Image Patches	176 (70%)	38 (15%)	38 (15%)
**Cell Classification**			
Syncytiotrophoblast	3212 (71%)	679 (15%)	646 (14%)
Cytotrophoblast	975 (71%)	207 (15%)	193 (14%)
Syncytial Knot	980 (72%)	175 (13%)	210 (15%)
Extravillus Trophoblast	1067 (74%)	201 (14%)	178 (12%)
Fibroblast	2516 (72%)	484 (14%)	491 (14%)
Hofbauer Cell	221 (72%)	40 (13%)	45 (15%)
Vascular Endothelial	1565 (72%)	304 (14%)	307 (14%)
Vascular Myocyte	1736 (70%)	370 (15%)	380 (15%)
Mesenchymal Cell	218 (67%)	58 (18%)	47 (15%)
Maternal Decidua	198 (69%)	47 (16%)	44 (15%)
Leukocyte	792 (69%)	158 (14%)	202 (18%)
**Tissue Classification**			
Villus Sprout	15545 (50%)	9066 (29%)	6441 (21%)
Terminal Villi	159464 (50%)	84052 (27%)	72402 (23%)
Mature Intermediate Villi	80954 (51%)	39296 (25%)	37332 (24%)
Stem Villi	132138 (55%)	58658 (25%)	48568 (20%)
Anchoring Villi	5769 (64%)	1634 (18%)	1654 (18%)
Chorionic Plate	46584 (84%)	4942 (9%)	3937 (7%)
Basal Plate and Septum	13329 (75%)	1029 (6%)	3359 (19%)
Fibrin	14195 (54%)	7047 (27%)	5010 (19%)
Avascular Villi	891 (49%)	526 (29%)	392 (22%)

Number of nuclei, cells, and tissue nodes used for training, validation and test sets of respective models followed by, in brackets, the proportion of data for that class across splits.

Supplementary Methods 1-2

Supplementary Method 1: Standard Operating Procedure provided to pathologists for validation task. The following text was provided to pathologists who participated in the human validation task. The task's purpose was to analyse inter-pathologist agreement across different tissue microstructures in 180 images, to contrast these agreement scores with model performance, and to compare pathologist majority agreement to ground truth annotations.

Placenta Human Validation SOP

Purpose

To assess the levels of agreement/disagreement between the author as a self-taught but untrained annotator (*original annotator*) to those of practising perinatal pathologists on H&E stained placenta histology substructures. Substructures of interest are split into two categories: tissues and other large structures (i.e. terminal villus or areas of fibrin) and cells (i.e. syncytiotrophoblast).

Each participating pathologist will be anonymous and will not be able to see the choices or interactions of other pathologists throughout the validation process. No data which can be linked to any one individual will be shared.

Ultimately, the original annotator's annotations will be used to train and validate a deep learning approach to modelling placenta substructures. Indirectly, the inputs gathered from perinatal pathologists will form part of this validation for the deep learning models.

Setup/Design

Whole Slide Image and Patient Selection

The whole slide image chosen for validation is an H&E stained parenchyma section of a term placenta from an uncomplicated pregnancy, without adverse maternal or neonatal outcomes. Pathologist's notes from the delivery site write that this section is histologically normal and "inflammation free… with villi corresponding to gestational age".

Substructures of Interest

**Tissue Structures**	**Cells**
Terminal Villus	Syncytiotrophoblast
Mature Intermediary Villus	Cytotrophoblast
Immature Intermediary Villus	Fibroblast
Stem Villus	Hofbauer Cell
Anchoring Villus	Vascular Endothelial Cell

Mesenchymal Villus	Vascular Myocyte
Villus Sprout	Maternal Decidual Cell
Chorion/Amnion	Extra Villus Trophoblast
Avascular Villus	Leukocyte
Basal Plate/Septa	Mesenchymal Cell
Fibrin	
Inflammatory Response	

Substructure References:

- https://link.springer.com/chapter/10.1007/978-3-030-11425-1_36
- https://www.proteinatlas.org/learn/dictionary/normal/placenta
- https://www.cambridge.org/core/books/placental-and-gestational-pathology/normal-development/11AA85DC1FF68EDA1165A2B46102F1D2
- https://onlinelibrary.wiley.com/doi/10.1111/apm.12858

Software Used

Original annotations and images were made using QuPath v3.1 (https://qupath.github.io/), images and collected data are stored securely in the Google Cloud Platform (https://cloud.google.com/), and the human validation interface is built using LabelBox (https://labelbox.com/).

Tissue Validation Process

Each pathologist is invited to join the LabelBox project. After signing in, they are presented with an image containing a centred single tissue structure or a cluster of assumed same-type tissue structures. They have several options per image, they can:

- view a polygon highlighting the area of interest (see image)
- set the tissue structure label and submit it
- say the tissue structure is unclear from the image and skip it
- say the tissue structure isn't in the list of options
- add a text comment to the image

Once they have submitted their label choice, they are presented with the next image in the queue. Images are queued such that all pathologists are shown the same images in the same order and the queue contains an even, but random, distribution of each tissue class originally annotated on the term sample. The exact number of each tissue class is not disclosed to avoid biasing the pathologists.

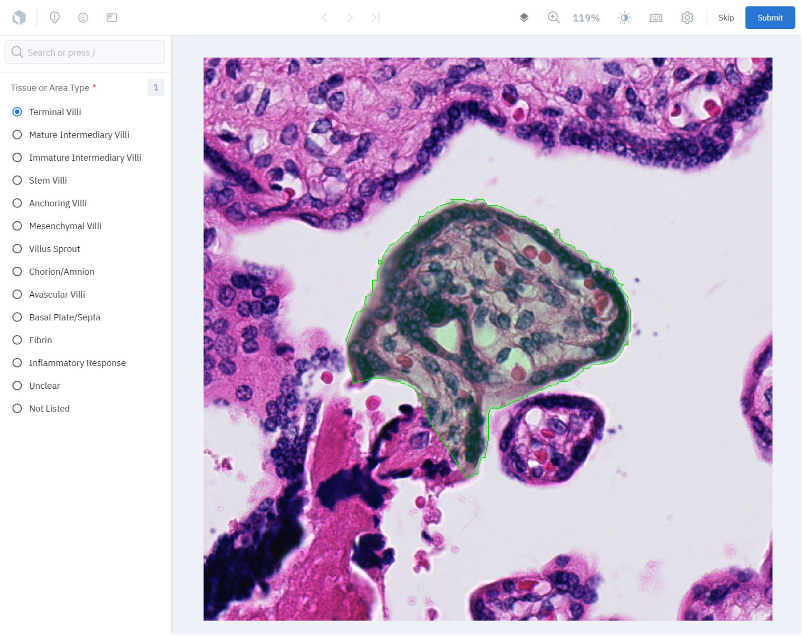

Tissue Image Generation

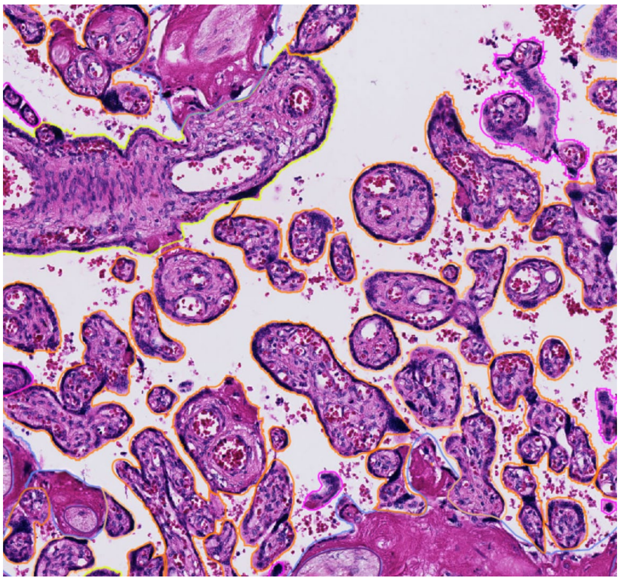

1. Polygons around structures were drawn using QuPath's Pixel Classifier and then corrected by the original annotator.
2. Tissue structure classes were assigned by the original annotator.
3. A downsampled image of the tissue boundaries was extracted so that each image contained a centred tissue or a cluster of same-type tissues.
4. For the larger structures such as the chorion, multiple images were instead extracted from smaller regions.
5. The corresponding coordinates of the polygons were saved in a json file.
6. Duplicate images with overlaid polygons were generated.
7. Images and polygon images were uploaded to Google Cloud and linked to LabelBox.

Data Collected for Validation

- Chosen tissue structure label
- Time spent on each image
- Number of images submitted
- Number of images submitted as 'unclear' or 'not listed'
- Optional comments submitted by pathologists
    - Comments will remain anonymous and will not be shared or quoted publicly

Downstream Analysis

The data collected from pathologist validators will be used to assess the untrained original annotator. The annotator's performance will be judged by the amount of agreement between their original annotations and the pathologists' label choices. The agreement will be quantified using Cohen's Kappa across all labelled data and also individually for each tissue type.

Agreement between pathologists will be quantified using the same metrics to assess the relative difficulty of the task. Of particular interest is whether there are any consistently difficult tissue structures that have low agreement scores and whether there are structures that have common patterns of disagreement (i.e. is there high disagreement between terminal villi and mature intermediary villi, for example).

These patterns of disagreement, should they exist, will also be used as a benchmark against the performance of a deep learning model on the same task. For example, if there is a large disagreement between terminal villi and mature intermediary villi by pathologists, then we would expect a deep learning model to mistake the two tissues more commonly than it would terminal villi and stem villi.

Supplementary Method 2: Validation tutorial provided to pathologists. The following text was provided to pathologists who participated in the human validation task as a tutorial for using the labelling software.

Placenta Tissue Validation Guide

Thank you for taking the time to validate these placenta tissues!

Instructions:

- You will be shown images containing tissue structures.
- To see the original image without overlay, use the ‘Image overlay’ button and select ‘Layer 2’.
- Choose a tissue structure from the list on the left and ‘Submit’ the image.
- If you are unsure, change the tissue type to ‘Unclear’ and ‘Submit’.
- You may optionally leave a comment on the image.

Further instructions on how to **show the original image**, **change the tissue type**, **see placenta sample information**, and **add a comment** are shown in the pages that follow.

If you would like a reference guide to **villus tissue structures** from the literature, see the final page.

Show Original Image

Click on the ‘Image overlay’ button in the top right and then select ‘layer 2’. You may use ‘layer 1’ to return the polygon overlay.

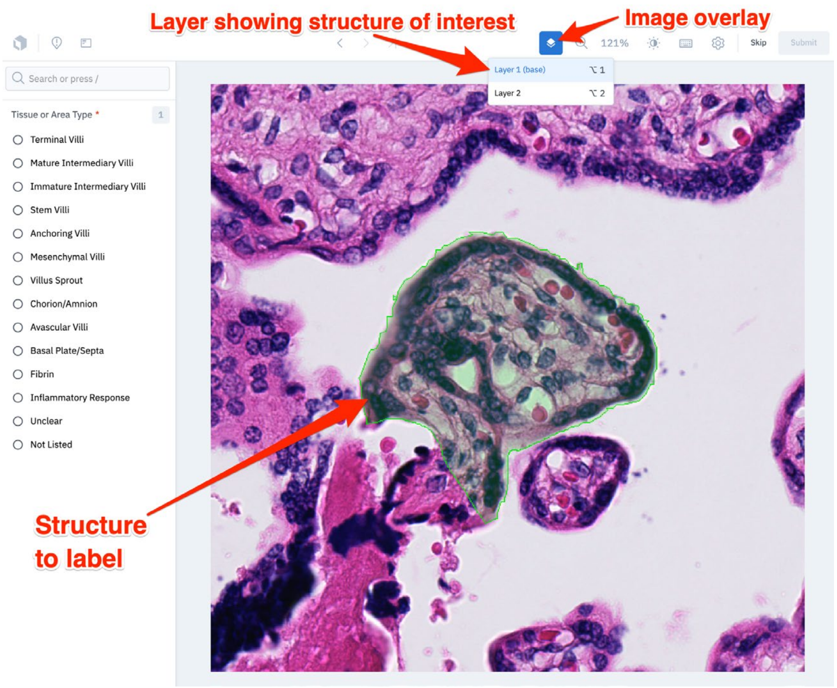

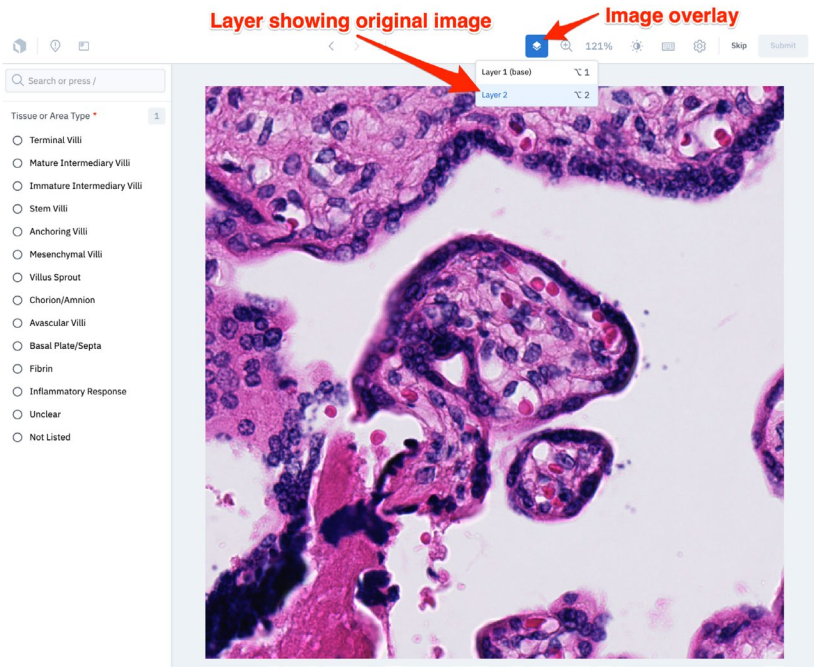

Change the Tissue Type

Choose an option from the list on the left and then press the 'Submit' button in the top right.

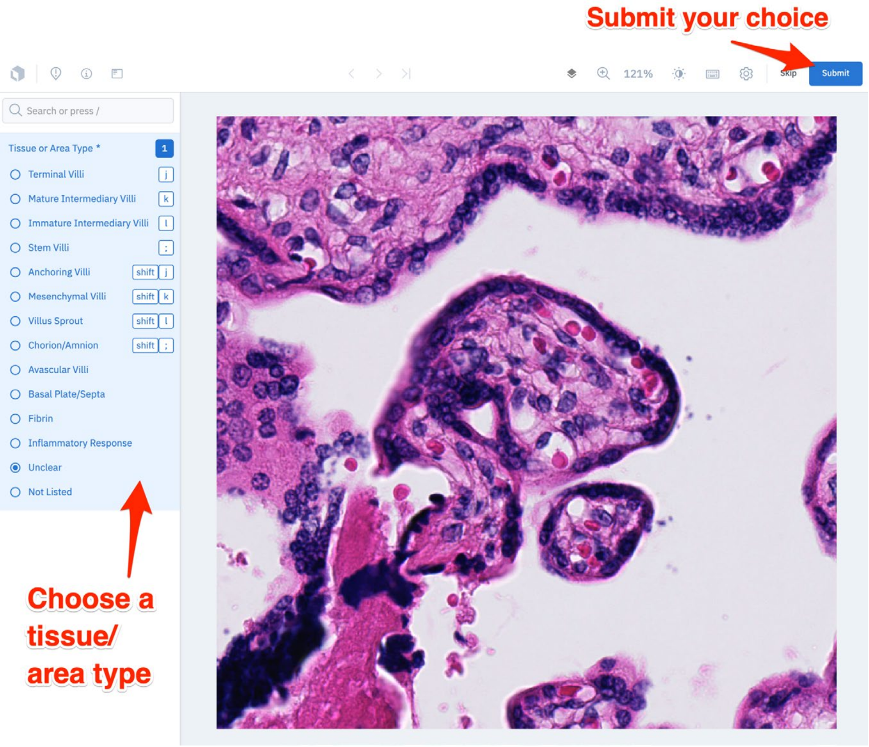

See Placenta Sample Information

Press the 'Info' icon at the top left to see information about the placenta.

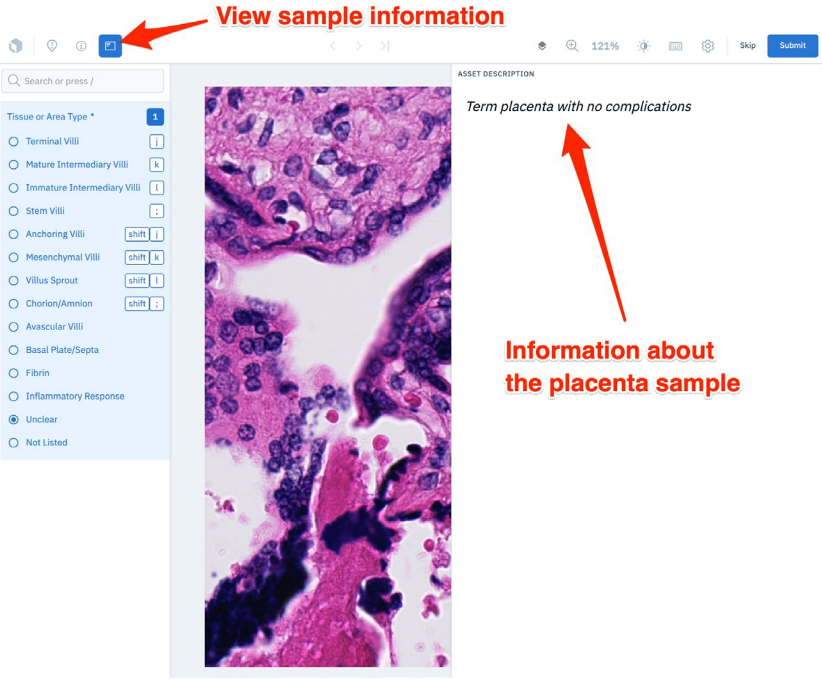

Add a Comment (Optional)

Press the ‘Issue’ button at the top left, click on the image, and write a comment in the box on the right.

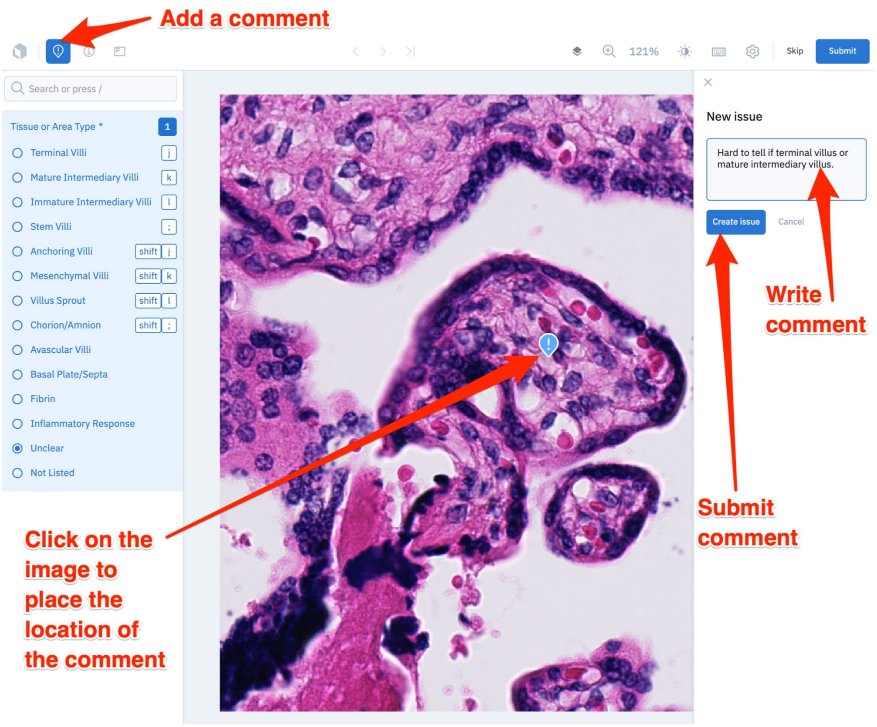

Tissue Structure Reference Guide

Literature:

1. https://link.springer.com/chapter/10.1007/978-3-030-11425-1_36
   - A comprehensive chapter detailing normal placental development with histology examples and villus tissue diagrams
2. https://onlinelibrary.wiley.com/doi/10.1111/apm.12858
   - A summary of placental maturity disorders with histology examples and villus tissue diagrams
3. https://www.cambridge.org/core/books/placental-and-gestational-pathology/normal-development/11AA85DC1FF68EDA1165A2B46102F1D2
   - A summary of normal development which focuses on changes across gestational age
4. https://www.proteinatlas.org/learn/dictionary/normal/placenta
   - An interactive healthy, term placenta histology slide with some example tissue types
5. http://eknygos.lsmuni.lt/springer/343/121-173.pdf
   - A pdf link of Benirschke’s *Architecture of normal villous trees* Chapter from *Pathology of the Human Placenta*

Supplementary References

1. Ernst, L. M. & Carreon, C. K. Placenta. in *Color Atlas of Human Fetal and Neonatal Histology* (eds. Ernst, L. M., Ruchelli, E. D., Carreon, C. K. & Huff, D. S.) 399–424 (Springer International Publishing, 2019). doi:10.1007/978-3-030-11425-1_36.
2. Benirschke, K., Burton, G. J. & Baergen, R. N. Architecture of Normal Villous Trees. in *Pathology of the Human Placenta* (eds. Benirschke, K., Burton, G. J. & Baergen, R. N.) 101–144 (Springer, 2012). doi:10.1007/978-3-642-23941-0_7.
3. Buslaev, A., Parinov, A., Khvedchenya, E., Iglovikov, V. I. & Kalinin, A. A. Albumentations: fast and flexible image augmentations. *Information* **11**, 125 (2020).
4. Ruifrok, A. C. & Johnston, D. A. Quantification of histochemical staining by color deconvolution. *Anal. Quant. Cytol. Histol.* **23**, 291–299 (2001).
