# Supplementary material for: Mapping cell-to-tissue graphs across human placenta histology whole slide images using deep learning with HAPPY
Source: Nat Commun. 2024 Mar 28;15:2710. doi: 10.1038/s41467-024-46986-2 (PMC10978962; doi:10.1038/s41467-024-46986-2)
Supplement: Supplementary file 2 — Reporting Summary [file 41467_2024_46986_MOESM2_ESM.pdf]

nature portfolio

Corresponding author(s): Christoffer Nellåker, Claudia Vanea

Last updated by author(s): Jan 27, 2024

Reporting Summary

Nature Portfolio wishes to improve the reproducibility of the work that we publish. This form provides structure for consistency and transparency in reporting. For further information on Nature Portfolio policies, see our Editorial Policies and the Editorial Policy Checklist.

Statistics

For all statistical analyses, confirm that the following items are present in the figure legend, table legend, main text, or Methods section.

n/a	Confirmed	
☐	☒	The exact sample size (*n*) for each experimental group/condition, given as a discrete number and unit of measurement
☒	☐	A statement on whether measurements were taken from distinct samples or whether the same sample was measured repeatedly
☐	☒	The statistical test(s) used AND whether they are one- or two-sided<br>*Only common tests should be described solely by name; describe more complex techniques in the Methods section.*
☐	☒	A description of all covariates tested
☐	☒	A description of any assumptions or corrections, such as tests of normality and adjustment for multiple comparisons
☐	☒	A full description of the statistical parameters including central tendency (e.g. means) or other basic estimates (e.g. regression coefficient) AND variation (e.g. standard deviation) or associated estimates of uncertainty (e.g. confidence intervals)
☐	☒	For null hypothesis testing, the test statistic (e.g. *F*, *t*, *r*) with confidence intervals, effect sizes, degrees of freedom and *P* value noted<br>*Give P values as exact values whenever suitable.*
☒	☐	For Bayesian analysis, information on the choice of priors and Markov chain Monte Carlo settings
☒	☐	For hierarchical and complex designs, identification of the appropriate level for tests and full reporting of outcomes
☒	☐	Estimates of effect sizes (e.g. Cohen's *d*, Pearson's *r*), indicating how they were calculated

Our web collection on statistics for biologists contains articles on many of the points above.

Software and code

Policy information about availability of computer code

Data collection	Hamamatsu XR, 3D HISTECH PANNORAMIC 250 Flash III and Aperio GT 450 scanners were used for whole slide image data collection. QuPath (v0.3.1) was used to create training datasets for each deep learning model.
Data analysis	We used Python version 3.10.13. The following Python packages available at the Python packaging index (PyPI, https://pypi.org/) were used for analysis and model training: pyvips 2.1.14, openslide-python 1.2.0, python-bioformats 4.0.7, PyTorch 2.0.1, torchvision 0.15.2, PyTorch Geometric 2.3.1, albumentations 1.3.0, peewee 3.16.2, pytest 7.3.1, typer 0.9.0, visdom 0.2.4, matplotlib 3.7.1, pandas 2.0.1, numpy 1.24.1, scikit-image 0.22.0, scikit-learn 1.3.1, umap-learn 0.5.3, seaborn 0.12.2. For reading whole slide images we used the C libraries libvips 8.9.2 and OpenSlide 3.4.1 via their python bindings listed previously. Source code is available at: https://github.com/Nellaker-group/happy

For manuscripts utilizing custom algorithms or software that are central to the research but not yet described in published literature, software must be made available to editors and reviewers. We strongly encourage code deposition in a community repository (e.g. GitHub). See the Nature Portfolio guidelines for submitting code & software for further information.

Data

Policy information about availability of data

All manuscripts must include a data availability statement. This statement should provide the following information, where applicable:

- Accession codes, unique identifiers, or web links for publicly available datasets
- A description of any restrictions on data availability
- For clinical datasets or third party data, please ensure that the statement adheres to our policy

The datasets generated for training and validating each deep learning model along with trained model weights are available for download at the Google Drive link: https://tinyurl.com/happyplacenta or from Zenodo: 10.5281/zenodo.10535021 with no restrictions. Instructions can be found in the GitHub readme at: https://github.com/Nellaker-group/happy. The two histology slides used for graph model training are available for download under CC BY 4.0 from the BioImage Archive at 10.6019/S-BIAD1045. The remaining in-house placenta histology slides and clinical data are not made available in accordance with existing research ethics committee approvals and data transfer agreements. Pretrained ImageNet weights for the RetinaNet and ResNet-50 models were downloaded in code via PyTorch from https://download.pytorch.org/models/resnet101-5d3b4d8f.pth and https://download.pytorch.org/models/resnet50-0676ba61.pth and will be downloaded programmatically on first model use. Source data are provided with this paper.

Research involving human participants, their data, or biological material

Policy information about studies with human participants or human data. See also policy information about sex, gender (identity/presentation), and sexual orientation and race, ethnicity and racism.

Reporting on sex and gender	No sex- and gender-based analysis have been performed. The study involved retrospective analysis of placenta histology slides which were collected during routine clinical care. Sex and gender are not currently believed to be clinically relevant for placenta pathology health assessment and so this information was not available to us in the provided clinical data.
Reporting on race, ethnicity, or other socially relevant groupings	No race- and ethnicity-based analysis have been performed. Race and ethnicity are not available to us in the provided clinical data.
Population characteristics	Relevant population characteristics of our in-house placenta histology slides is summarized in Supplementary Table 4 and Supplementary Table 5. As described in the manuscript, we report findings for 'healthy' term placentas and those which had clinically significant placental infarction. All slides were assessed using pathology reports generated at each institute by a perinatal pathologist and for the placentas with infarction, all slides were selected to contain a region of the infarction.
Recruitment	Patients were not directly involved or recruited. The study involved retrospective analysis of placenta histology slides which were collected during routine clinical care.
Ethics oversight	Ethical approval was obtained from the Research Ethics Committee of the University of Tartu (289/T-5), the Helsinki Committee at the Hadassah Medical Center (0735-18-HMO), and an exemption for approval was obtained from the institutional review board at the Northshore Health System (EH23-303) under the condition that clinical data, beyond information that slides were histologically normal term placentas, was not shared.

Note that full information on the approval of the study protocol must also be provided in the manuscript.

Field-specific reporting

Please select the one below that is the best fit for your research. If you are not sure, read the appropriate sections before making your selection.

☒ Life sciences ☐ Behavioural & social sciences ☐ Ecological, evolutionary & environmental sciences

For a reference copy of the document with all sections, see nature.com/documents/nr-reporting-summary-flat.pdf

Life sciences study design

All studies must disclose on these points even when the disclosure is negative.

Sample size	No sample size calculations were performed. We used all available in-house placenta histology slides which satisfied our selection criteria. We determined sufficient sample size by increasing the quantity of nuclei (n=11,755), cell (n=13,842), and tissue (n=468,869) training data until asymptotic improvement of model performance was achieved.
Data exclusions	Beyond selection for healthy term placentas and those with placental infarction no data was excluded from this work
Replication	Findings may be replicated using our publicly available software and released datasets. Restrictions apply to the availability of the placenta histology in-house data, which were used with rigorous ethical and legal approval by local ethics boards for the current study, and are thus not publicly available. Findings across placenta whole slide images may be replicated by running our software on other slides.
Randomization	For nuclei detection and cell classification model training, our datasets were randomly split into 70/15/15% training, validation, and test images. For graph tissue classification model training, a healthy term slide from the University of Tartu was randomly selected to contain

training, validation, and test node splits with an additional randomly selected slide from the Hadassah Medical Center containing further training nodes. To mitigate the risk of information leakage across node datasets when randomly split nodes are part of the same neighbourhood, we split nodes into validation and test sets across large regions in the slide. Validation and test node regions were chosen such that they were larger than the 16-hop neighbourhood aggregation distance and contained a similar annotated distribution of tissue classes as the distribution across the whole slide. See Supplementary Fig. 8 for a visualization of these regions. For the 'Comparison to perinatal pathologists' analysis, 180 tissues evenly split into tissue types were randomly selected from the full annotated set. For the 'Quantitative metrics for placental health' and 'A Case Study of Placental Infarction' analysis, all model-unseen healthy term placenta slides and slides with clinically significant placental infarction were used.

Blinding

Blinding was not applicable as our experiments involved retrospective analysis of placenta histology whole slide images.

Reporting for specific materials, systems and methods

We require information from authors about some types of materials, experimental systems and methods used in many studies. Here, indicate whether each material, system or method listed is relevant to your study. If you are not sure if a list item applies to your research, read the appropriate section before selecting a response.

Materials & experimental systems

n/a	Involved in the study
☒	☐ Antibodies
☒	☐ Eukaryotic cell lines
☒	☐ Palaeontology and archaeology
☒	☐ Animals and other organisms
☒	☐ Clinical data
☒	☐ Dual use research of concern
☒	☐ Plants

Methods

n/a	Involved in the study
☒	☐ ChIP-seq
☒	☐ Flow cytometry
☒	☐ MRI-based neuroimaging

Plants

Seed stocks

Report on the source of all seed stocks or other plant material used. If applicable, state the seed stock centre and catalogue number. If plant specimens were collected from the field, describe the collection location, date and sampling procedures.

Novel plant genotypes

Describe the methods by which all novel plant genotypes were produced. This includes those generated by transgenic approaches, gene editing, chemical/radiation-based mutagenesis and hybridization. For transgenic lines, describe the transformation method, the number of independent lines analyzed and the generation upon which experiments were performed. For gene-edited lines, describe the editor used, the endogenous sequence targeted for editing, the targeting guide RNA sequence (if applicable) and how the editor was applied.

Authentication

Describe any authentication procedures for each seed stock used or novel genotype generated. Describe any experiments used to assess the effect of a mutation and, where applicable, how potential secondary effects (e.g. second site T-DNA insertions, mosiacism, off-target gene editing) were examined.
